# Manipulating Myc for reparative regeneration

**DOI:** 10.3389/fcell.2024.1357589

**Published:** 2024-03-21

**Authors:** Camilla Ascanelli, Rowda Dahir, Catherine H. Wilson

**Affiliations:** Department of Pharmacology, University of Cambridge, Cambridge, United Kingdom

**Keywords:** MYC, regeneration, cell cycle, proliferation, repair

## Abstract

The Myc family of proto-oncogenes is a key node for the signal transduction of external pro-proliferative signals to the cellular processes required for development, tissue homoeostasis maintenance, and regeneration across evolution. The tight regulation of Myc synthesis and activity is essential for restricting its oncogenic potential. In this review, we highlight the central role that Myc plays in regeneration across the animal kingdom (from Cnidaria to echinoderms to Chordata) and how Myc could be employed to unlock the regenerative potential of non-regenerative tissues in humans for therapeutic purposes. Mastering the fine balance of harnessing the ability of Myc to promote transcription without triggering oncogenesis may open the door to many exciting opportunities for therapeutic development across a wide array of diseases.

## 1 Myc structure, function, and the proximal Myc network

Myc belongs to a class of proto-oncogenes (comprising *c-Myc*, *n-Myc*, and *l-Myc*), which are genes whose product induces cell proliferation in response to mitogenic stimuli and that can become oncogenic upon their mutation or deregulation (Vennstrom et al., 1982). While much Myc research has focused on its oncogenic properties, its activities as a proto-oncogenic transcription factor positions Myc as a key downstream factor in many signal transduction pathways important for development, tissue homoeostasis, and regeneration (such as WNT, RAS/RAF/MAPK, JAK/STAT, TGF-β, and NF-κB) (Dang, 2012). As such, it is part of the proximal Myc network (PMN), a system of transcription factors that consolidates signals from several distinct upstream pathways into the expression of thousands of target genes involved in many biological processes (Grandori et al., 2000; Conacci-Sorrell et al., 2014).

**FIGURE 1:**
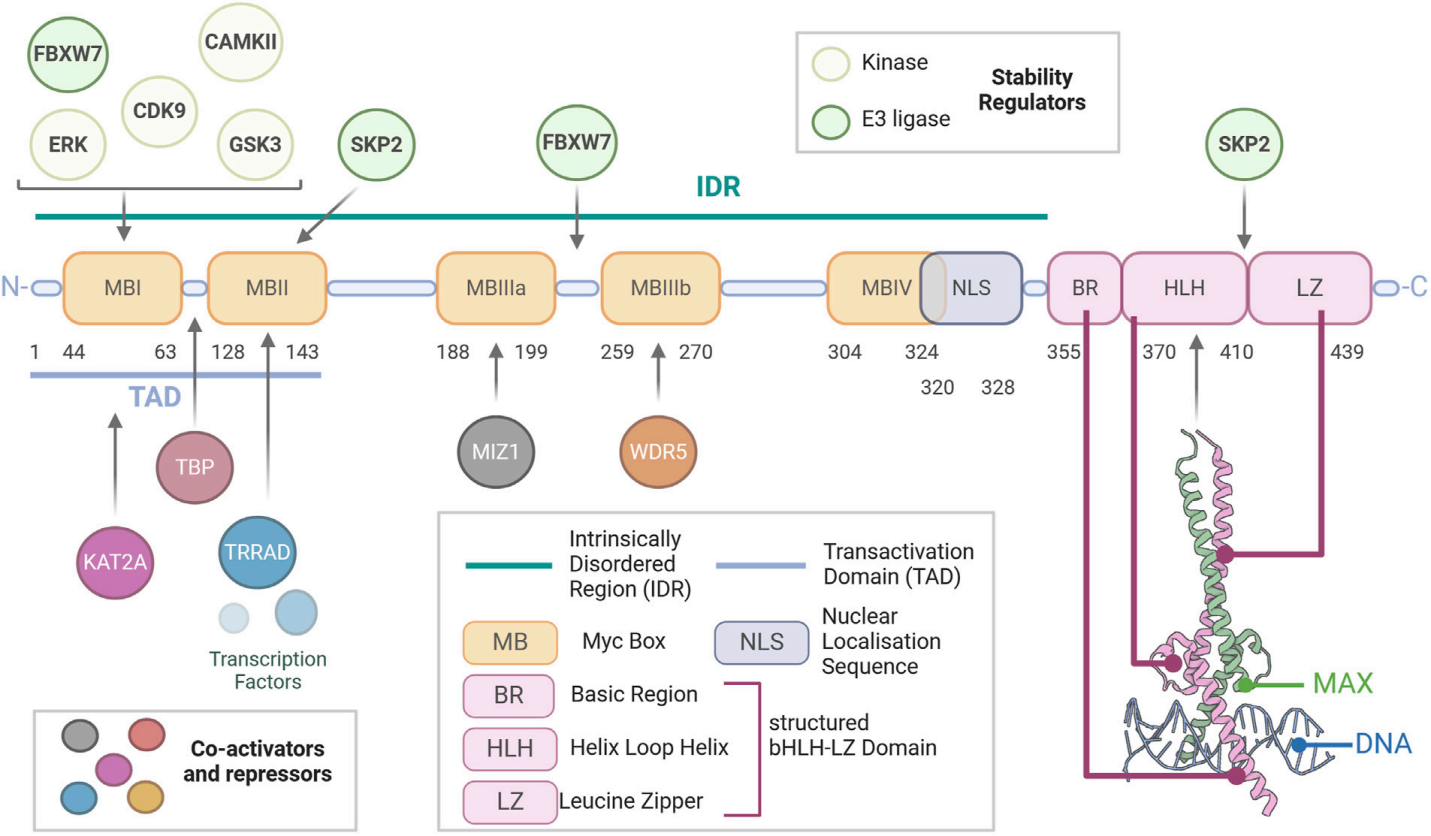
Structure of Myc. Schematic representation of the modular amino acid sequence of the Myc transcription factor. Myc is a largely unstructured protein with a vast intrinsically disordered region (IDR) extending from the N-terminus to the beginning of its basic region. The IDR contains the nuclear localisation sequence (NLS) and multiple disordered regions called Myc boxes (MB), which are conserved between Myc family members and are key to the function of Myc, being sites of essential protein–protein interactions. Most importantly, the transactivation domain (TAD), which contains MBI and MBII, allows for the binding of the co-activators and repressors of Myc activity. Within this, MBI is also a binding site for most regulators of Myc stability. Finally, essential for the ability of Myc to elicit transcription, the basic helix–loop–helix leucine zipper (bHLH-LZ) domain is essential for binding of Myc to MAX and the DNA, as shown in the crystal structure (PDBID:1NKP).

All members of the Myc family are dimerizing transcription factors that contain a basic helix–loop–helix leucine zipper (bHLH-LZ) domain (Figure 1). The heterodimers can interact with the DNA through recognition of an enhancer box (E-box, 5′-CACGTG-3′) via the bHLH-LZ; this drives the recruitment of co-activators/repressors, transcriptional regulation, and chromatin remodelling. The bHLH-LZ domain is present on the carboxyl-terminus (C-terminus) of Myc and has been shown to have helical conformation when unbound; it only assumes its full structure when bound to MAX and the DNA (Nair and Burley, 2003; Sammak et al., 2019). The amino-terminus (N-terminus) consists of a large unstructured intrinsically disordered region (IDR) containing multiple conserved domains called Myc boxes (MB). MBs are sites of interaction with regulators and interactors (transactivation domain—TAD, comprising MBI-MBII) and degron motifs central to Myc degradation (Sears et al., 1999; Sears et al., 2000; Sears, 2004). Importantly, Myc is unable to homodimerise and cannot bind DNA as a monomer, thus requiring its obligatory partner MAX (Amati et al., 1992, 1993). Due to its lack of functional domains, MAX does not possess direct transcriptional activity but rather forms transcriptionally inactive complexes in the form of MAX homodimers and heterodimers with MAX-binding proteins and dimerization proteins (e.g., MNT, MGA, MAD1-4, and MXI1), becoming functional antagonists to Myc-MAX dimers by competing for E-box binding. Therefore, MAX is the central node of the PMN, whereby changes in the balance between its heterodimerisation partners determine cell fate decisions and a switch from a proliferative or transformative state when Myc is abundant to differentiation or quiescence when abundant MADs outcompete Myc (Grandori et al., 2000; Conacci-Sorrell et al., 2014).

The overarching function of Myc in healthy tissues is to integrate multiple signals derived from different pathways to elicit global transcriptional change. The transcriptional activity of Myc hinges on its ability to recruit RNA polymerase II and members of histone acetylase complexes to Myc-binding sites, with Myc target sites presenting high histone acetylation. Specifically, the region between MBI and MBII binds the TATA-binding protein (TBP), a member of the transcription factor IID (TFIID) complex responsible for recruiting RNA polymerase II (RNAPII) at transcriptional start sites (Wei et al., 2019). Through MBI, Myc recruits the cyclin T1-CDK9 complex, which together comprise the positive transcription factor B (P-TEFb) that elicits phosphorylation of RNAPII and releases it from transcriptional pausing, thus initiating transcriptional elongation (Rahl et al., 2010). The abundance of P-TEFb is rate-limiting to Myc-driven hyper-transcription (Bywater et al., 2020). MBII mediates Myc’s interaction with other regulators of transcriptional activity, including transformation/transcription domain-associated protein (TRRAP), an adaptor protein that forms complexes with lysine (K) acetyltransferase (KATs). MBIIIb interacts with WDR5 (WD repeat domain 5), an essential component of H3K4 methyltransferase complex (Couture et al., 2006; Thomas et al., 2015). Finally, Myc possesses transcriptional repressor activity, which MBIIIa mediates, specifically through interaction with MIZ-1, a transcriptional activator if not bound to Myc. MIZ-1’s binding to co-activators p300 and NPM1 is impeded in the Myc-MIZ-1 bound form (Vousden, 2002; Möröy et al., 2011).

**FIGURE 2:**
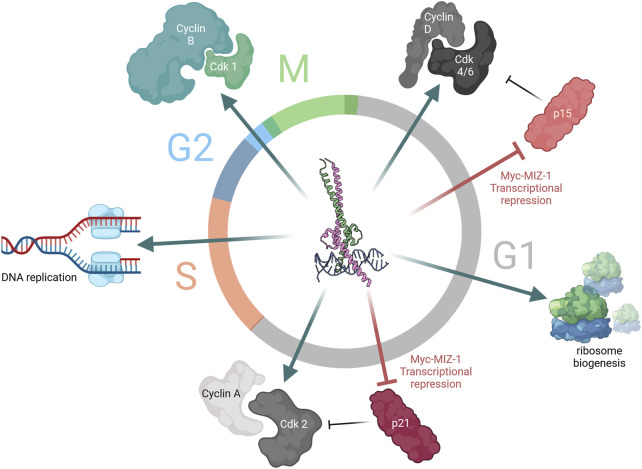
Myc is a key driver of cell cycle progression. Myc-driven cell cycle progression is ubiquitous throughout the different stages of the cell cycle. Early in G1, expression of cyclin D and cyclin-dependent kinase (Cdk) 4/6 is driven by Myc-MAX upon mitogenic sensing, concomitant with the repression of cyclin-dependent kinase inhibitor (CDKI) p15 by Myc-MIZ-1. Similarly, later in G1, the role of Myc as transcriptional activator and repressor continues to induce transcription of cyclin A and Cdk2 and repress CDKI p21 expression. Meanwhile, Myc also drives ribosome biogenesis through upregulation of RNA Pol III and tRNA expression, coupling cell cycle progression with increasing cellular size. In S-phase, Myc participates in DNA replication. Finally, at the G2/M transition, Myc induces the expression of the mitotic cyclin, cyclin B1.

The result of Myc-driven transcription is the amplified expression of genes involved in various cellular programmes including proliferation, apoptosis (Evan et al., 1992; Kanazawa et al., 2003), metabolism (Stine et al., 2015), and senescence (Hydbring and Larsson, 2010; Singh et al., 2023). Myc-driven cell cycle progression results from its combined function as a transcriptional amplifier and repressor, with Myc mRNA and protein levels closely correlating with proliferation rates (Kelly et al., 1983; Dean et al., 1986; Waters et al., 1991; Bretones et al., 2015). Myc has been shown to directly bind components of the pre-replicative complex (Pre-RC), necessary for DNA replication; in early G1 phase, it binds the origin recognition complex, located at the origin of replication (Dominguez-Sola and Gautier, 2014). Activation of Pre-RCs to induce the functional initiation of transcription requires cyclin-dependent kinase (CDK) activity. Myc directly induces the expression of cyclins and cyclin-dependent kinases, specifically cyclins A, B, and D, as well as Cdk-4 and Cdk-6 (Bretones et al., 2015; García-Gutiérrez et al., 2019). As mentioned above, MIZ-1-bound Myc is capable of transcriptional repression, with two known targets of Myc-MIZ-1’s transcriptional repression being p21Cip1 and p15Ink4b—two cyclin-dependent kinase inhibitors (CDKIs). For both genes, the Myc-MIZ-1 heterodimers bind the transcriptional start site, which does not affect the basal-level expression of these genes but, rather, their induction by anti-mitotic stimuli (Vousden, 2002; Wiese et al., 2013). Myc also prevents CDKI expression/activity through indirect mechanisms whereby it increases Cdk1 levels which phosphorylates p27, leading to degradation by E3 ligase Skp2 (García-Gutiérrez et al., 2019). Myc activity promotes ribosome biogenesis by regulating the expression of the core subunits of the RNA polymerase I apparatus and interacting directly to enhance pre-rRNA processing. Myc enhances the transcription of RNA polymerase III subunits, with which it cooperates to yield 5S RNA and tRNA production (Campbell and White, 2014). Furthermore, Myc-induced transcriptional amplification results in the upregulation of genes involved in nucleotide and miRNA synthesis, enzymes involved in RNA processing and capping, and eukaryotic translational initiation factor 4E (eIF4E) (Stine et al., 2015), allowing Myc to modulate cellular transcription. Myc is essential for sustained proliferation and rate-limiting for cell cycle progression, with cells which express high levels of Myc progressing to S-phase more rapidly than lowly expressing cells which present a longer G_0_/G_1_ (Liu et al., 2023). Furthermore, the inhibition of Myc expression in a panel of human cancerous and non-cancerous cell lines consistently results in cell cycle arrest (Wang et al., 2008). Interestingly, the cell cycle phase at which cell lines arrest varies according to their background, with healthy cells exiting the cell cycle at G_0_/G_1_, while most cancer cell lines displayed an arrest in later stages (S or G_2_/M) (Wang et al., 2008). Altogether, Myc is essential for cell cycle progression where its contribution is three-fold: coupling cell growth with cell cycle progression, repressing cell cycle inhibitor proteins, and inducing DNA replication, transcription, and translation (Figure 2).

## 2 Control of Myc activity for safeguarding tissue integrity

**FIGURE 3:**
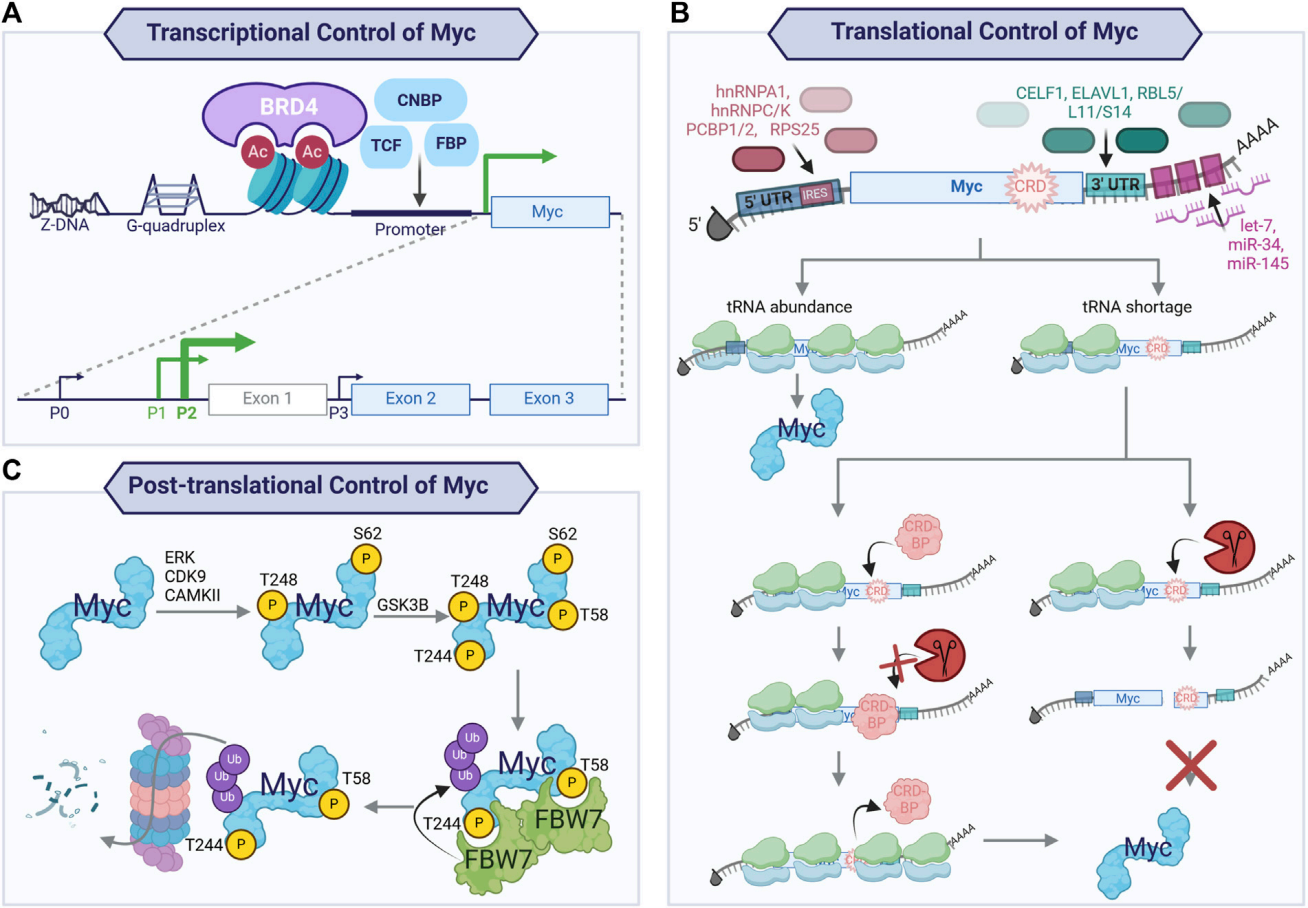
Transcriptional, translational, and post-translational control of Myc. Tight control of Myc expression, translation, and protein half-life is exacted to maintain physiological levels of Myc in regenerative tissues. **Transcriptional control of Myc** **(A)** is achieved through non-B DNA structures (Z-DNA and G-quadruplexes), binding of transcription factors (CNBP, TCF, FBP), and BET domain-containing transcriptional regulator, BRD4. This yields transcription preferentially from two of the four promoters (P0, P1, P2, and P3), with the majority of transcripts arising from P2 and, to a lesser extent, P1. The mRNA arising from P2 and P1 consists of three exons, with exons 2 and 3 encoding the main Myc protein isoform. The mRNA of the proto-oncogene is also subject to tight **translational regulation (B)**, resulting in a short-lived mRNA. The transcripts generated from P2 and P1 encode for a long 5′UTR which contains independent ribosome entry sites (IRES) providing binding sites for RNA-binding proteins (RBP; hnRNPA1, hnRNPC, hnRNPK, PCBP1, PCBP2, and RPS25). The coding sequence contains a coding region instability determinant (CRD) which, in the context of tRNA codon shortage, will cause ribosomal stalling and endonucleolytic attack by an endonuclease if not protected by a CRD-binding protein (CRD-BP). At the 3′UTR of Myc mRNA, CELF1 and ELAVL1 compete for binding to balance the transcriptional output, with CELF1 decreasing ELAVL1 association with the mRNA and, therefore, decreased transcriptional output. Additionally, the RNA-induced silencing complex (RISC) is recruited to Myc mRNA by ribosomal proteins (RB) L5, L11, S14, and miRNA binding at the 3′ UTR. Finally, the Myc protein is highly unstable with a short half-life, due to its many destabilising protein–protein interactions. Illustrated here **(C)** is the key mechanism for **Myc protein turnover** via a series of post-translational modifications. Myc is bound and phosphorylated by kinases (e.g., ERK, CDK9, and CAMKII) at S62 and likely T248, providing a priming phosphorylation that allows for GSK3β binding. This kinase phosphorylates T58 (and probably T244), generating phosphodegron sites for E3 ligase FBW7 binding. Once bound, dimers of FBW7 can ubiquitinate Myc, leading to its degradation by the ubiquitin proteasome pathway.

To safeguard against the impact of the activation of the proto-oncogene on promoting cell proliferation, multiple processes converge to restrain Myc levels and activity. Therefore, Myc is highly regulated at the transcriptional, translational, and post-translational levels (Figure 3). The *Myc* gene is located within an approximately 3-Mb area of chromosome 8q24 that lacks protein-coding genes. *Myc* expression is regulated by a wide array of transcription factors, including CNBP, FBP, and TCF (Levens, 2010), and by BRD4, a BET domain-containing transcriptional regulator (Delmore et al., 2011; Mertz et al., 2011). Additionally, non-B DNA structures are involved in regulating *Myc* expression: Z-DNA, single-strand bubbles, and G-quadruplexes, which are tertiary structures formed by guanine-rich sequences that are present in the NHEIII region of the Myc promoter (Brooks and Hurley, 2009; Brooks and Hurley, 2010; Levens, 2010). This region of chromosome 8 contains tissue-specific long-range enhancers and super-enhancers of *Myc* that contribute to modulating *Myc* expression (Lancho and Herranz, 2018).

The *Myc* gene contains three exons, with exons two and three encoding the protein and transcription arising predominantly from promoters P1 (25%) and P2 (75%) (Liu and Levens, 2006; Wierstra and Alves, 2008). Myc mRNA arises from different splicing of the three exons, and the resulting mRNA possesses a short half-life. Multiple microRNAs, such as let-7, miR-34, and miR-145, can target it for degradation (Sampson et al., 2007; Kim et al., 2009; Sachdeva et al., 2009; Cannell et al., 2010; Kress et al., 2011). Ribosomal proteins L5, L11, and S14 also bind *Myc* at the 3′ UTR, leading to its degradation by the RNA-induced silencing complex (RISC) via miR-24 (Liao et al., 2014; Spiniello et al., 2019) and miR-145 (Zhou et al., 2013). *Myc* mRNA contains a coding region instability determinant (CRD) region with rare codons that cause destabilisation of the mRNA upon ribosomal stalling, thus hindering translation when not protected from endonucleolytic attack by a CRD-binding protein (CRD-BP, also known as insulin-like growth factor II mRNA-binding protein-1 (IGF2BP1)). Levels of CRD-BP are high in the foetus but decrease to low or absent in adult life (Leeds et al., 1997; Lemm and Ross, 2002; Weidensdorfer et al., 2009; Spiniello et al., 2019), allowing rapid *Myc* mRNA turnover in adult tissues. The untranslated regions (UTRs) of *Myc* mRNA are sites for regulation by RNA-binding proteins (RBPs). The long 5′ UTR arising from P1 and P2 promoters contain internal ribosomal entry sequences (IRESs) which interact with RBPs such as hnRNPC, hnRNPK, PCBP1, PCBP2, hnRNPA1, and RPS25 (Kim J. H. et al., 2003; Evans et al., 2003; Audic and Hartley, 2004). CELF1 and ELAV1 (also named HuR) compete to bind the 3’ UTR, resulting in a balance of translational output, with CWLF1-binding resulting in decreased association of *Myc* mRNA with ELAVL1, thus reducing its translation (Liu et al., 2015; Spiniello et al., 2019). Many of these interactors were recently confirmed by HyPR-MS (hybridization purification of RNA–protein complexes followed by mass spectrometry—Spiniello et al., 2019). Concomitantly, novel RBPs ranging in function were identified, such as histone variant, transcription and translation factors, structural constituents of the spliceosome, nuclear ribonucleoproteins, and proteins involved in nuclear export mechanisms and mRNA metabolism (Spiniello et al., 2019); this demonstrates the complex regulation to which *Myc* mRNA is subject.

Once translated, the Myc protein is subject to tight post-translational control, resulting in a short-lived protein whose half-life is ∼15–30 min. The most well-characterised pathway for Myc protein degradation results in the phosphorylation of phosphodegrons that allow the recognition by E3 ligase FBW7 (F-box/WD repeat-containing protein 7), a member of the SCF (SKP1, CUL1, and F-box proteins) complex. Specifically, the phosphorylation of serine 62 (S62) is involved in Myc stabilisation upon mitogen sensing and cell cycle re-entry and has been shown to be catalysed by ERK as part of the RAS/RAF/MAPK signalling cascade, amongst others (Sears et al., 1999; Sears et al., 2000; Sears, 2004). Phosphorylation at S62 is a pre-requisite for phosphorylation at threonine 58 (T58) by glycogen synthase kinase 3β (GSK3β). S62 and T58 phosphorylation occurs at different times of the cell cycle. Upon mitogen sensing and cell cycle entry, the RAS/RAF/MAPK signalling cascade is activated, leading to Myc phosphorylation and stabilisation, and inhibition of GSK3β via the activation of the PI(3)K/Akt signalling pathway, thus promoting early accumulation of pS62 Myc. Later in the G1 phase, Akt activity declines, leading to increased GSK3β activity, raising the levels of the double-phosphorylated form of Myc, and overall destabilising Myc, thus increasing its turnover. Recent evidence has shown that multiple kinases (ERK (Sears et al., 2000; Marampon et al., 2006; Hayes et al., 2016; Vaseva et al., 2018), CDK9 (Blake et al., 2019; Hashiguchi et al., 2019), and CAMKII (Gu et al., 2017)) phosphorylate Myc at S62 and pharmacological inhibition of such kinases can lead to decreased Myc protein stability. Subsequent to the phosphorylation of both S62 and T58, a series of interactions results in Myc with a single phosphorylated T58—the phosphodegron motif recognised by Myc’s main E3 ligase, FBW7. A second phosphodegron site for FBW7 has recently been identified at T244 and T248 (Welcker et al., 2022). According to these findings, in the context of over-expressed Myc, FBW7 monomers can recognise either of the phosphodegron sites, leading to the ubiquitination and degradation of Myc. Indeed, ablation of phosphodegron at T58 via an alanine mutation (T58A), which had been previously reported as a version of Myc non-degradable by FBW7, was bound and degraded by FBW7 during the phosphorylation of T244 and T248. Conversely, in the context of endogenous Myc, both phosphodegrons are needed to allow FBW7 dimers to bind and degrade Myc. Other E3 ligases have also been shown to degrade Myc, especially Skp2, whose ubiquitination of Myc not only causes its degradation but also increases its transcriptional activity as it acts as a transcriptional co-activator (Kim S. Y. et al., 2003).

Finally, to safeguard against deregulated levels of Myc that bypass its transcriptional, translational, and post-translational control, Myc activity can trigger apoptosis in non-malignant cells (Wyllie et al., 1987; Evan et al., 1992; Murphy et al., 2008). The balance between the proliferative and proapoptotic activity of Myc depends on its transcriptional control and the cellular context in which it is activated as the proapoptotic response of Myc can be dependent on p53 activation (Zindy et al., 1998). It is important to highlight that Myc-induced proliferation and apoptosis are governed by distinct thresholds and are largely thought to be caused by Myc's ability to engage the same set of target genes, modulating the degree of target gene transcription in different cellular contexts. Therefore, in most cells, modest Myc activation can lead to increased proliferation and transformation and also to the low-level expression of proapoptotic genes. However, in cells already primed for apoptotic response and lacking other oncogenic lesions, Myc can trigger proliferation but will also amplify the proapoptotic response, leading to both p53-dependent and -independent cell death (Murphy et al., 2008; McMahon, 2014; Jha et al., 2023).

## 3 Myc and regeneration across evolution

**FIGURE 4:**
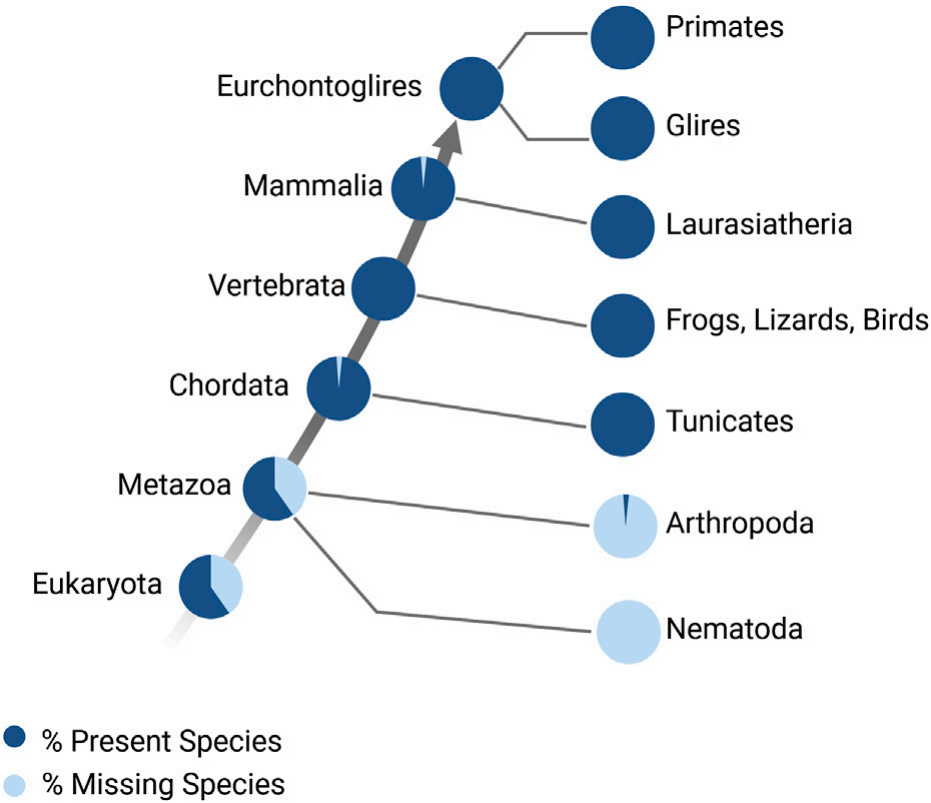
Myc family of genes over evolution. Percentage of species that have Myc family genes (Accession: TF106001), modified from http://www.treefam.org/ (Li, 2006; Li, 2006).

The ability of Myc to orchestrate cell cycle re-entry and proliferation makes Myc crucial for tissue regeneration. At its simplest level, regeneration is the regulation of transcription to drive proliferation and differentiation (cell fate changes) which lead to renewal or restoration of tissue function. The regenerative ability of tissues and organs varies widely across the animal kingdom, from whole-body regeneration in hydras to complex organ (e.g., limb, heart) regeneration in zebrafish and salamanders; more limited regeneration is observed in mammalian species, and regeneration is often limited to certain tissues at certain times (Yun, 2015). The Myc family of genes arose very early during evolution, before the diversification of metazoan evolution (Figure 4) (Young et al., 2011; Mahani et al., 2013); however, across species, the basic biochemical properties of Myc are very highly conserved. Therefore, the function of Myc as a master transcriptional regulator and its role in regeneration has been extensively studied across regenerative models.

*Hydra* live in fresh water and are members of the phylum Cnidaria. The species is one of the earliest to have evolved complex tissues in a defined body plan (Reddy et al., 2019), and they can regenerate their entire body following transverse and longitudinal dissection and dissociation. Four homologues of Myc (myc1, myc2, myc3, and myc4) and *Hydra*-max have been identified in Hydra; biochemically, myc and max complex and bind to E-Boxes. Once bound to DNA, the proteins transcriptionally regulate genes, such as cad, leading to cell cycle progression and ribosome biogenesis (Hartl et al., 2010; Young et al., 2011). *In situ* hybridization and single-cell RNA sequencing expression analysis have determined that the Hydra myc1 and myc2 genes are localised in all the proliferative cells of the animal, including the continuously proliferating interstitial cells, proliferating epithelial stem cells throughout the gastric region, and epithelial cells during gametogenesis. Myc3 lacks the N-terminal Myc boxes and is exclusively expressed in progenitor cells committed to nerve and gland cell differentiation (Hartl et al., 2010, 2014; Lechable et al., 2023). Myc expression is absent in all terminally differentiated cell types such as nerve cells and nematocytes (Young et al., 2011; Lechable et al., 2023). Myc has been shown to be crucial for controlling cell proliferation and differentiation processes in *Hydra*. It has been hypothesized that the myc1 and 2 homologues may compete with myc3 for max and E-boxes and regulate proliferation and differentiation, presumably by interacting with different protein partners, given the difference in the TADs (Lechable et al., 2023). Importantly, RNAi-mediated knockdown of myc1 during injury impairs the equilibrium between stem cell self-renewal and differentiation, leading to abnormal tentacle morphogenesis (Ambrosone et al., 2012). This suggests that Myc plays a key role in *Hydra* regenerative mechanisms (Table 1).

**TABLE 1:** Role of Myc across regenerative species.

Species	Organ	Uninjured localisation of Myc	Injured localisation of Myc	Perturbation	Author
*Hydra*	Whole animal	Interstitial stem cells	No significant changes in expression	RNAi-mediated knockdown during injury results in abnormal tentacle morphogenesis	Hartl et al. (2010)
		Nematoblast nests			Lechable et al. (2023)
		Gland cells			Ambrosone et al. (2012)
*Hothuria glaberrima* (sea cucumber)	Digestive tube	Luminal epithelium of the intestine	Extensively in the mesothelial epithelial cells at 3 days post-injury	RNAi-mediated knockdown during injury results in reduced cell proliferation in intestinal explant	Mashanov et al. (2015a)
		Scattered individual cells in the mesothelium			Quispe-Parra et al. (2021a)
					Mashanov et al. (2015b)
	Radial nerve cord	Apical regions of the neuroepithelia	Apical regions of the neuroepithelia	RNAi-mediated knockdown during CNS injury results in failure of radial glial activation and dedifferentiation	Mashanov et al. (2015a)
		Scattered cell bodies in the neural parenchyma	Glial tubes		Quispe-Parra et al. (2021b)
			Radial nerve cords		Mashanov et al. (2015b)
*Polyandrocarpa misakiensis* (sea squirt)	Bud development	Proximal half of the developing bud		RNAi-mediated knockdown results in defects in gut formation	Fujiwara et al. (2011)
		Atrial epithelium			
		Branchial and gut primordia			
		Mesenchyme cells near the organ primordia			
*Ambystoma mexicanum* (Axolotl)	Limb	Little or undetected	Blastema at 3 h to 3 days		Stewart et al. (2013), Géraudie et al. (1989)
			Wound epidermis		
			Mesenchymatous-like cells		
*Xenopus laevis* froglet (African clawed frog)	Limb and tail	Low but present in the growing froglet limb	Wound epithelium		Géraudie et al. (1990)
			Limb regenerate		Lemaître et al. (1992), Christen et al. (2010)
			Mesenchymal cells in the blastema		
			Regenerating tail bud		
			Notochord		
			Neural tube		
*Podarcis muralis* (Wall lizard)	Tail	Sparse or undetected	Regenerative blastema		Alibardi (2017)
			Basal layers of the apical-lateral wound epidermis		Degan et al. (2021)
			Mesenchymal-like cells		
*Danio rerio* (zebrafish)	Retina	Low or undetected	Pan-retinal at 12 h post-injury	Morpholino knockdown or pharmacological Myc inhibitor blocks cell proliferation and Muller glia reprogramming in the retina	Mitra et al. (2019)
			Muller glia-derived progenitor cells and adjacent cells		
			Ganglion cell layer		
	Neuromast hair cell	Undetected	Supporting cells within the boundary of mantle cells	Myc inhibition with small molecule or peptide reduces the number of regenerated hair cells	Lee et al. (2016)
	Fin		Pharmacological Myc inhibitor blocks fin regeneration	Mitra et al. (2019)

Another regenerative phylum is the invertebrate Echinoderms, which includes starfish, sea urchins, and sea cucumbers. Quite remarkably, the sea cucumber, *Holothuria glaberrima,* can regenerate most of its internal and external organs, following injury (García-Arrarás et al., 2018); even major parts of its central nervous system (CNS) can renew following severe injury. The sea cucumber homologue of Myc, like other species, contains a bHLH-LZ and TAD. Characterisation of *H. glaberrima* Myc expression in both the intestine and CNS immediately following injury demonstrates that Myc expression levels sharply increase, and both organs undergo extensive cell dedifferentiation (Mashanov et al., 2015a). This suggests that Myc is a critical transcription factor involved in the immediate regenerative response. Furthermore, a correlation is observed between increased Myc expression and the expression of genes involved in ribosomal biogenesis at the first and third days after intestinal injury (Quispe-Parra D. J. et al., 2021). The functional role of Myc in *H. glaberrima* regeneration has been determined by RNAi-mediated knockdown of Myc during injury. Knockdown of Myc during intestinal explant regeneration leads to reduced cell proliferation with no effect on dedifferentiation (Quispe-Parra D. et al., 2021). In the CNS, Myc denial at the same time as injury leads to a failure in radial glial activation, dedifferentiation, and a decrease in cellular apoptosis (Mashanov et al., 2015b). Together, these results indicate that Myc is a key gene controlling the immediate proliferative regenerative response in *H. glaberrima,* while the effect on dedifferentiation may be context-specific.

Ascidians or sea squirts are marine invertebrate sessile tunicates that belong to phylum Chordata. Ascidians present with a single Myc gene that contains a bHLH-LZ (Vanni et al., 2022). In ascidian species *Botryllus schlosseri*, *Ciona savignyi*, and *Polyandrocarpa misakiensis*, Myc is expressed in early development and disappears in adult tissues (Kobayashi et al., 2022; Vanni et al., 2022). Knockdown of Myc in embryonic/larval stages via morpholinos (modified antisense oligonucleotides), RNAi, or a dominant negative version of Myc suppresses mesenchymal and endodermal cell cycle and impairs organogenesis (Fujiwara et al., 2011; Kobayashi et al., 2022).

Amphibian species such as *Ambystoma mexicanum* (axolotl) and the African clawed frog, *Xenopus laevis*, have varying regenerative capacities. Axolotls remain in their juvenile stage throughout life and can regrow limbs and multiple internal organs, including the brain, spinal cord, liver, skeletal muscle, heart, and eyes. In contrast, *X. laevis* loses much of its regenerative ability when they metamorphose from tadpoles to adult frogs. There is surprisingly little research into the role of Myc in axolotl regenerative capacity; however, RNA sequencing data indicate that Myc is rapidly expressed at day 1 post-limb amputation and remains enriched for 10 days (Stewart et al., 2013). Proteomics data from limb amputation at days 1, 4, and 7 following injury highlight Myc as one of the most highly connected transcription factors (Jhamb et al., 2011); in agreement, regenerating axolotl limbs express Myc (Géraudie et al., 1989). There is a strong correlation in *X. laevis* between Myc expression and regeneration. In an undamaged setting, Myc expression in juvenile froglet limbs is low, but, following injury, Myc expression rapidly and significantly increases, together with the expression of proliferative marker PCNA. Myc expression then falls to a baseline by day 5 following resection (Géraudie et al., 1990; Lemaître et al., 1992; Christen et al., 2010). In reptiles (*Podarcis muralis*) after tail amputation, Myc expression has been studied by qRT-PCR, Western blotting, and immunohistochemical techniques and Myc is observed in the regenerating blastema in a similar location to the proliferating cells (Alibardi, 2017; Alibardi, 2022; Degan et al., 2021).

Zebrafish, *Danio rerio*, are teleosts (bony fish) which have been used as a regenerative model since the 1970s because of their incredible capacity to regenerate amputated fins, brain lesions, retinas, spinal cords, and hearts. Like mammals, the zebrafish Myc family consists of three family members—c-myc, N-myc, and L-myc—which complex with zebrafish max. The temporal and spatial expression patterns of Myc in zebrafish during development indicate that L-myc expression is limited to very early embryonic stages, whereas c-myc and N-myc are expressed during periods of growth and active cellular proliferation. N-myc expression is significantly downregulated in terminally differentiated adult tissues, whereas c-myc expression persists in some adult tissues such as gills and liver (Schreiber-Agus et al., 1993). The role of Myc has been studied across zebrafish regenerative organs, and several lines of evidence across cell types suggest that Myc is essential for an appropriate regenerative response. In the heart, the results from transgenic chemically induced cardiac injury and RNA sequencing have shown dramatic increases in Myc target gene expression, including genes involved in cell cycle and oxidative phosphorylation; this suggests a role for Myc in the induction of cardiomyocyte cell proliferation and mitochondrial biogenesis (Miklas et al., 2022). However, an alternative study using cardiac cryo-injury suggested that Myc target genes are downregulated at days 4 and 7 post injury, which is surprising given the observed increase in G2M checkpoint gene expression—which would normally overlap with Myc targets (Dicks et al., 2020). In the zebrafish retina, Myc expression is transiently upregulated following retinal injury, appearing 1 h post-injury, peaking at 24 h, and remaining increased for 7 days post-injury. Increased Myc expression coincides with elevated proliferative markers PCNA and BrdU and regulates the dedifferentiation of Muller glia to Muller glia-derived progenitor cells. Knockdown of Myc using morpholinos or the blockade of the Myc–Max interaction using the pharmacological inhibitor 10058-F4 abolishes proliferation and Muller glia reprogramming in the retina (Mitra et al., 2019). Another regenerative system in zebrafish is the sensory hair cells in the inner ear. During neuromast hair cell regeneration following damage, sensory hair cells display a rapid upregulation of Myc at 1 h that drops back to baseline levels by 18 h. The inhibition of Myc with 10058F4 or a cell-permeable Myc-specific peptide inhibitor suppresses cell cycle re-entry and hair cell regeneration (Lee et al., 2016). Furthermore, 10058-F4, abolishes fin regeneration (Mitra et al., 2019), demonstrating that Myc is essential to several regenerative processes in Zebrafish.

## 4 Myc and regeneration in mammals

Mammals have a more limited regenerative ability than amphibians and fish. In mammals, tissue regeneration processes are often classified into physiological regeneration and reparative regeneration. Ongoing physiological regeneration includes organs such as the intestinal gut lining, skin epidermis, red blood cells, and endometrium, whereby homoeostatic cell replacement involves stem cell differentiation or the replication of existing cells by proliferation or trans-differentiation (Iismaa et al., 2018). Reparative regeneration involves the restoration of damaged tissue or lost body parts and is therefore triggered by injury. Examples of organs that can partially or completely regenerate in adult mammals are the liver, spleen, bone, peripheral nerve, and urinary bladder (Mehta and Singh, 2019).

The role of Myc in maintaining tissue homoeostasis was first reported in pancreatic β-cells where it is activated in response to elevated levels of plasma glucose (Yamashita et al., 1988; Jonas et al., 2001), suggesting that it plays a role in β-cell proliferation and tissue maintenance under physiological conditions. Myc is also transiently expressed at days 1 and 2 during pancreatic regeneration after subtotal pancreatectomy in rats (Calvo et al., 1991). However, the function of Myc has best been characterised in the context of hyperglycemia, where Myc is shown to lead to altered secretory function and loss of differentiation of β-cells. Other reports of the role of Myc in β-cells showed that it is not necessary for the functioning of adult β-cells in physiological conditions but plays a key role in maintaining tissue homoeostasis in young mice under metabolic stress, whereby knockout of Myc in mouse β-cells resulted in β-cell dysfunction and impaired glucose tolerance. This protective function of Myc was shown to be lost in ageing mice, possibly through hypomethylation of the Myc response element (Rosselot et al., 2019). Interestingly, when Myc overexpression was explored as a therapeutic option to rescue dysfunctional β-cells, Myc induced cell death and differentiation (Laybutt et al., 2002; Pelengaris et al., 2002; Cheung et al., 2010). The observed cell death may be due to the overexpression methods chosen as a previous study demonstrated that the expression of Myc in β-cells from two different promoters resulted in β-cell proliferation or apoptosis, depending on the low- or high-expression system, respectively. Indeed, when the expression of Myc was driven from the locus that most accurately reproduced physiological levels of the proto-oncogene, Myc-induced apoptosis was only recorded in islets upon treatment with a sub-apoptotic dose of doxycycline (Murphy et al., 2008).

The involvement of Myc in the homoeostasis and wound healing of many epithelial tissues has been well-documented. Myc plays a vital role in the maintenance and regeneration of mammalian intestinal crypts. In a physiologically normal setting, Wnt-signalling in the rapidly proliferating progenitor and amplifying cells of the intestinal crypts drives Myc expression. Once the cells move out of the crypt niche and travel up the intestinal villi, they stop proliferating, Myc expression is lost, and the cells become terminally differentiated. The process of epithelia turnover takes around 5 days. Although Myc is not essential for the homoeostatic maintenance of juvenile and adult intestines (Bettess et al., 2005; Muncan et al., 2006; Konsavage et al., 2012), Myc null progenitor cells are smaller in cell size, have slowed cell cycle progression, reduced biosynthetic activity, and result in smaller daughter cells (Muncan et al., 2006). In a regenerating setting, following damage with gamma irradiation, Myc plays a vital role in the repair of intestine crypts. Wnt and c-Myc signalling is activated during intestinal regeneration (Muncan et al., 2006; Ashton et al., 2010) and where Myc is conditionally deleted, Myc-null crypts do not regenerate, and intestines become completely denuded of crypts. Therefore, Myc plays a central role in the regenerating intestine (Ashton et al., 2010).

The research surrounding the role of Myc in skin homoeostasis is complex and context dependent. Knockout of Myc in the basal cells within the epidermis of mice reveals that keratinocytes can continue to cycle, suggesting that Myc is not necessary for cell division, but animals display defects such as tight and fragile skin (Zanet et al., 2005). Conversely, others have shown that Myc is dispensable for epidermal homoeostasis and that mice show no defects in skin phenotypes (Oskarsson et al., 2006). Studies overexpressing Myc in epidermal cells have found that Myc can trigger proliferation and disrupt the differentiation of postmitotic keratinocytes (Pelengaris et al., 1999). However, others have determined that Myc overexpression can stimulate differentiation rather than drive proliferation (Gandarillas and Watt, 1997). To reconcile these opposing findings, it has been proposed that the level, duration, and timing of Myc may determine whether cells enter a proliferative or terminal differentiation state (Watt et al., 2008). In an injury setting following skin epidermal wounding, Myc levels significantly increase 7 days post-injury, co-localising with the proliferative marker BrdU. The levels of Myc then remain high during wound closure, decreasing to near baseline levels by day 30 when wound healing is complete (Shi et al., 2015). Denial of Myc during the wound healing process results in reduced proliferation in healing fronts and impaired healing with fewer layers of keratinocytes (Zanet et al., 2005). A recent eloquent study using lineage tracing and single-cell sequencing showed that wounding stimulates Myc-dependent dedifferentiation (Bernabé-Rubio et al., 2023).

Further evidence of the importance of Myc in epithelial regeneration in mammals comes from the oesophagus and lungs. The basal cells of the oesophageal epithelium express Myc relatively homogenously in an undamaged setting and require Myc for their self-renewal capacity. Upon conditional knockout of Myc (c-Myc and n-Myc), basal cells lose their undifferentiated state, leading to senescence (Hishida et al., 2022). In the lung, the conditional deletion of Myc in the epithelial club cells does not affect epithelial regeneration after naphthalene-induced injury, while the loss of Myc from the mesenchymal parabronchial smooth muscle cells causes reduced Fgf10 expression, decreased proliferation, and significantly impaired airway epithelial regeneration (Volckaert et al., 2013).

The role of Myc in reparative tissue regeneration has been extensively studied in the liver. Epithelial cell turnover in the liver is slow and the hepatocytes are mainly quiescent, with an estimated less than 1 in 10,000 hepatocytes in mitosis at any point in time (Kopp et al., 2016). The level of Myc in the homoeostatic liver is very low. During regeneration, after partial hepatectomy in rodents, over a third of hepatocytes can be seen proliferating within 24–26 h, and liver mass is restored to normal in around a week (Kopp et al., 2016; Michalopoulos and Bhushan, 2021). Following partial hepatectomy, Myc is rapidly induced within hours of damage, and Myc expression is followed by an increase in proliferation (Thompson et al., 1986; SOBCZAK et al., 1989; Morello et al., 1990), indicating its important role in driving hepatic regeneration. Similarly, following ectopic acute overexpression of Myc (low or high level) in the liver, rapid cell cycle progression and proliferation are observed (Murphy et al., 2008; Bywater et al., 2020).

Interestingly, Myc ablation studies have indicated that hepatocytes are still capable of entering the cell cycle in the absence of Myc (Baena et al., 2005; Sanders et al., 2012), suggesting that Myc is not essential for hepatocyte proliferation. However, the inhibition of Myc using antisense oligomers or the ablation of Myc in the regenerating rodent liver following partial hepatectomy leads to a reduction of proliferating cells (Arora et al., 2000; Baena et al., 2005; Rodríguez et al., 2006). More recently, knockout of Myc and Mlx (Max-like protein, the key node of the Mlx network and part of the Myc extended network) in mice has indicated that Myc denial leads to changes in the expression of mRNA translation and energy metabolism, ultimately impeding the regenerative potential of hepatocytes (Wang et al., 2022).

**FIGURE 5:**
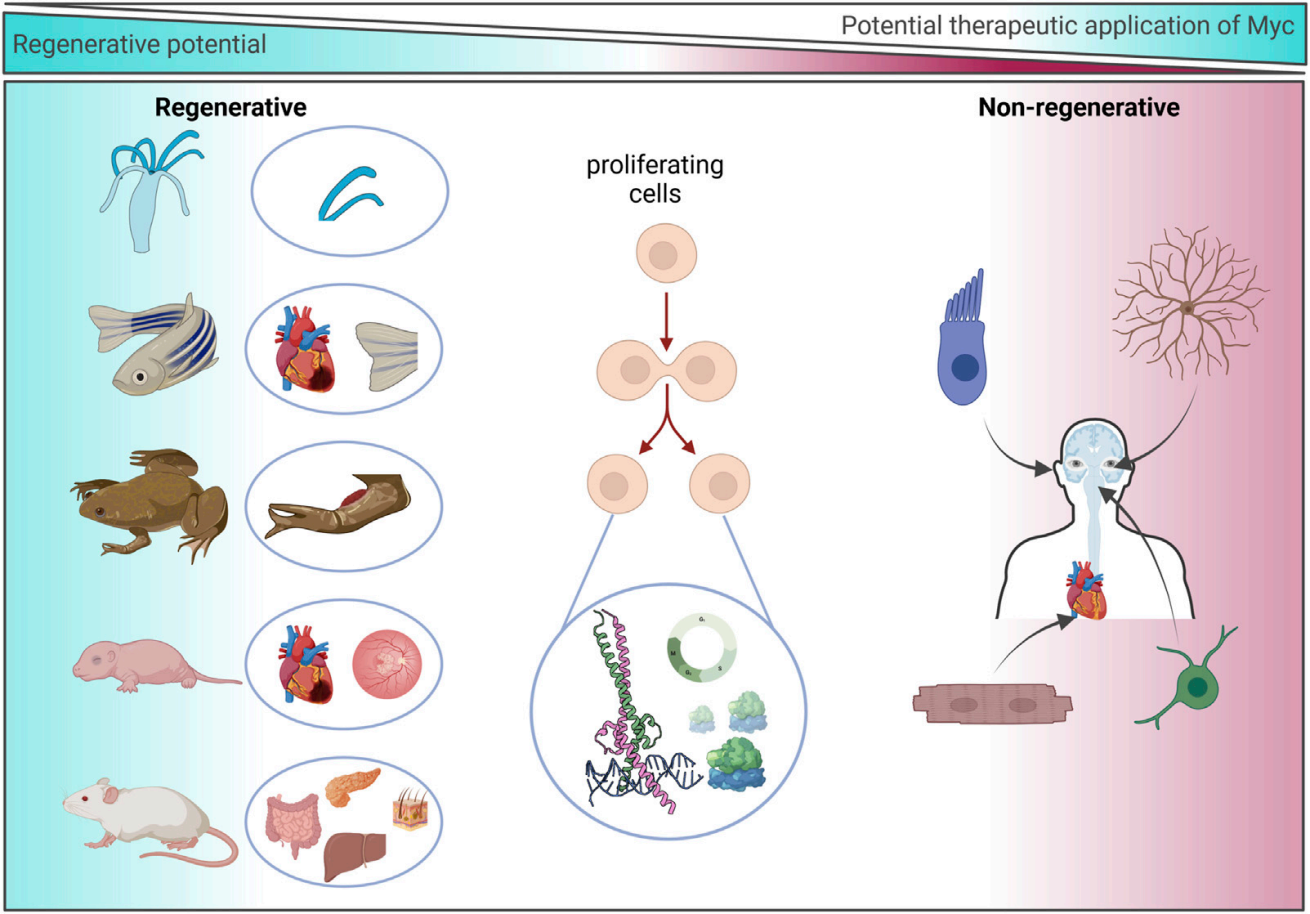
Regenerative potential of Myc. Representation of species/organs with an experimentally determined association or dependence upon Myc during regeneration (left) and non-regenerative human cell types in which the experimental use of Myc may be exploited to drive regeneration (right).

In general, data from regenerative species and regenerative tissues in mammals indicate that Myc is predominantly expressed in proliferating cells and that the expression of Myc drives key transcriptional programs, including ribosomal biogenesis, metabolism, and cell cycle progression. In normally quiescent but regenerative tissues following an insult, Myc expression can be observed as a short pulse, and its expression correlates with the pattern of proliferative cells. Myc appears to be non-essential to the homoeostatic regenerative processes of many organs, although, repair is attenuated when Myc is denied (Figure 5). Therefore, it is exciting to speculate whether Myc may have the potential to aid regeneration in tissues that do not normally have regenerative capacity.

## 5 Harnessing Myc in non-regenerative organs

Some adult mammalian tissues have strikingly little regenerative capacity, such as the heart and CNS. However, like *X. laevis*, some embryonic and neonatal mammal tissues have shown remarkable regenerative capacity. For instance, the adult mammalian heart cannot regenerate following injury, and loss of the contractile cardiomyocytes leads to adverse pathological remodelling that ultimately results in heart failure. Conversely, following resection of 15% of the myocardium at day 1 post-birth, the neonatal mouse heart can fully regenerate and regain normal cardiac function (Porrello et al., 2011). However, this regenerative ability is lost by day 7. Fate mapping has confirmed that regeneration occurs via cardiomyocyte proliferation, which is similar to the regenerative mechanism observed in the regenerating zebrafish heart (Porrello et al., 2011; Senyo et al., 2013). This short cardiac neonatal regenerative window has also been shown to exist in larger mammals such as pigs (Ye et al., 2018; Zhu et al., 2018), and there are some anecdotal case studies of newborn babies exhibiting a regenerative capacity briefly after birth (Haubner et al., 2016; Aly et al., 2021). Interestingly, the level of c-Myc and n-Myc in the mouse heart declines sharply at birth, and it is almost absent in the adult heart (Singh et al., 2018; Bywater et al., 2020). Transcriptional comparisons of the regenerating mouse neonatal heart to the adult non-regenerating heart indicate that adult cardiomyocytes do not express Myc and therefore fail to reactivate the neonatal transcriptional Myc programmes following injury (Quaife-Ryan et al., 2017; Singh et al., 2018). Even when Myc is specifically and ectopically activated in adult myocardium, the adult heart is refractory to proliferation (Xiao et al., 2001; Bywater et al., 2020; Chen et al., 2021). Myc instead induces hypertrophic growth and not hyperplasia, suggesting that Myc alone is insufficient for driving the cell cycle in cardiomyocytes (Xiao et al., 2001). However, global ChIP sequencing has established that Myc binds to largely overlapping promoter sites in proliferative (liver) and non-proliferative (heart) tissues that encode classic Myc programmes involved in ribosomal biogenesis and cell cycle, despite the difference in response to the activation of ectopic Myc. Interestingly, Myc-driven transcription in the heart is impeded by the limited availability of transcriptional machinery such as the P-TEFb complex, which allows efficient RNAPII-mediated transcriptional amplification (de Pretis et al., 2017; Bywater et al., 2020). Consequently, the Myc-driven transcriptional response is attenuated in cardiomyocytes, and, while many Myc target genes are seen to be marginally increased, hypertranscription is limited, leading to cell growth without division. Therefore, both Myc expression and Myc-driven transcription are limited in the adult mammalian heart. In agreement, Nox4 overexpression has been shown to prolong the postnatal period of cardiomyocyte proliferation via ERK1/2 activation and an increase in Myc phosphorylation. Stabilised Myc binds to and drives the expression of genes such as Cyclin D2, leading to cell cycle. However, Nox4 could not continue to drive cardiomyocyte proliferation in the adult heart, again indicating that Myc-driven proliferation is limited in an adult setting (Murray et al., 2015).

When Myc and the limiting transcriptional machinery, Cyclin T1, are ectopically expressed in cardiomyocytes, Myc-driven transcription is productive and can drive efficient cardiomyocyte proliferation with gene expression changes related to metabolism, cell proliferation, and division, and a reversion to the neonatal-like state (Singh et al., 2018; Bywater et al., 2020; Boikova et al., 2022). In an injury setting following experimental myocardial infarction, Myc together with Cyclin T1 overexpression, specifically in adult cardiomyocytes, can drive the functional repair of mouse hearts, so long as Myc expression is transient and localised to the injury site (Boikova et al., 2023). In an effort to develop a prototypical therapeutic to drive endogenous regeneration in the adult mouse heart, Myc and Cyclin T1 have been delivered via mRNA to drive a transient short pulse of Myc and Cyclin T1 expression. Despite the short expression time of the mRNA of less than 24 h, functional improvement over 28 days was observed, suggesting that Myc and Cyclin T1 could be harnessed to drive regeneration of the heart (Table 2). A number of matters remain to be resolved. For instance, mRNA was injected directly into the heart and will be expressed in multiple cell types; therefore, the therapeutic would be greatly enhanced by the use of cell-specific expression techniques (Magadum et al., 2020a; Magadum et al., 2020b), and a catheter-based delivery system would expand the target patient population considerably. Furthermore, the relative functional benefit observed from Myc-Cyclin T1 mRNA was lower than that from the transgenic systems, so extending the expression time of Myc and Cyclin T1 may enable greater reparative success. Finally, while neoplasia following Myc-Cyclin T1 mRNA expression was not observed over the course of the experiment, careful consideration of the oncogenic potential of Myc must be considered. Interestingly, forced expression of the reprogramming factors Oct4, Sox2, Klf4, and Myc (OSKM) has also been shown to re-program and drive cardiomyocyte proliferation, ameliorate myocardial damage, and drive functional improvement, following infarction. In this model, prolonged expression of OSKM causes cardiac teratoma formation, although short-term OSKM-induced cardiomyocyte dedifferentiation was shown to be reversible (Chen et al., 2021). Therefore, a system such as mRNA—which allows a more physiologically normal pulse of Myc expression as observed following acute damage in regenerative systems—which can be localized and has no issues surrounding genetic integration should be employed.

**TABLE 2:** Evidence of mammalian regeneration by Myc.

Species	Organ/cells	Ectopic overexpression technology	Injury model	Findings	Authors
Mouse	Heart	Tamoxifen-inducible MycER	No injury	Ectopic Myc and Cyclin T1 in adult and juvenile cardiomyocytes results in cardiomyocyte proliferation	Bywater et al. (2020) and Boikova et al. (2022)
		Tamoxifen-inducible MycER and modified mRNA-encoding Myc and Cyclin T1	Myocardial infarction by occlusion of the left anterior descending coronary artery	Transient and local expression of Myc with cyclin T1 around the infarct results in functional cardiac recovery and reduced scar size	Boikova et al. (2023)
Guinea pig	Cochlea	Adenoviral vector encoding Myc	Acoustic trauma	Smaller auditory threshold shift at 7-day post-noise exposure. Reduction in outer hair cell stereocilia loss and cilia disarray	Han et al. (2009)
Mouse	Ear/explant organ culture of the utricle	Adenoviral vector encoding MycT58A	No injury	Supporting cells re-enter the cell cycle and proliferate.	Burns et al. (2012a)
				Small number of cells differentiate towards the hair cell lineage	
Mouse	Ear/cochlea	Adenoviral vector encoding Myc	No injury	Combined transient ectopic Myc and Notch 1 intracelluar domain reprograms adult supporting cells to regenerate hair cell-like cells	Shu et al. (2019)
Mouse	Ear/cochlea	Cocktail of small molecules and siRNAs to activate Myc, Notch1, Wnt, and cAMP pathways	Kanamycin- and furosemide-induced hair cell loss	Reprograming of adult supporting cells to regenerate hair cell-like cells	Quan et al. (2023)
Mouse	Eye/optic nerve	Adeno-associated virus serotype 2 (AAV2) encoding Myc and tamoxifen-inducible MycER	Optic nerve injury by crushing	Increased survival of retinal ganglion cells and axonal regeneration following injury. Synergistic effects of ectopic Myc, PTEN, and SOC3 deletion	Belin et al. (2015)
Mouse	Eye/optic nerve	pEX4-c-Myc DNA plasmid	Optic nerve injury by crushing	Myc is both necessary and sufficient for sensory axon regeneration via the Myc-TERT-p53 signalling pathway	Ma et al. (2019)
Mouse	CNS/oligodendrocytes progenitor Cells (OPCs)	Dual-AAV system targeting *Pdgfra* endogenous locus resulting in ectopic Myc in all *Pdgfra*-expressing OPCs	No injury	Reprogramming of mature OPCs, increased OPC proliferation, and ability to differentiate into myelinating oligodendrocytes	Neumann et al. (2021)

The inner ear sensory hair cells are essential in detecting sound from the external environment. However, they lack the regenerative capabilities to replace damaged cells upon acoustic trauma, leading to permanent hearing loss. In contrast, lower invertebrates retain the ability to regenerate damaged hair cells by driving the proliferation of supporting cells (Corwin, 1981; Ryals and Rubel, 1988; Janesick et al., 2022). This ability has been shown to exist in neonatal mice, where supporting cells can re-enter the cycle and transdifferentiate into hair cells (White et al., 2006). It has been postulated in mouse utricles that 51% of hair cells that have arisen after birth are from proliferating supporting cells that transdifferentiate into hair cells (Burns et al., 2012a). In zebrafish, Myc has been shown to be essential for hair cell regeneration as it drives the proliferation of hair cell precursors (supporting cells) and is upregulated during the regeneration process (Lee et al., 2016). Both c-Myc and n-Myc have been found to be expressed in the mammalian inner ear during development, and n-Myc plays an essential role in morphogenesis, patterning, and proliferation during development (Domínguez-Frutos et al., 2011; Kopecky et al., 2011). The expression of n-Myc and c-Myc declines postnatally and they are expressed at low levels in the adult inner ear (Domínguez-Frutos et al., 2011). Myc has been shown to play a protective role against noise damage. Guinea pigs inoculated with an adenoviral vector-encoding Myc prior to exposure to noise damage had a smaller auditory threshold shift 7-days post-noise exposure. Furthermore, morphological assessment of the cochlea indicated that Myc expression reduced the outer hair cell stereocilia loss and cilia disarray. These results indicate that the ectopic expression of Myc reduces the loss of hair cells, following acoustic trauma (Han et al., 2009).

The overexpression of Myc in a cultured adult mouse utricle can reverse the quiescent and post-mitotic state of the supporting cells and allow them to re-enter the cell cycle and drive proliferation. Some of these cells have acquired the ability to differentiate towards the hair cell lineage by expressing the hair cell marker myosin VIIA (Burns et al., 2012b). Furthermore, Myc and Cyclin A2 were reported to be downregulated during cochlear development and the overexpression of both genes was shown to enhance the proliferation of cochlear progenitor cells (Zhong et al., 2015). *In vivo*, the combined over-expression of Myc and Notch 1 intracellular domain in the adult mouse inner ear drives the proliferation of supporting and inner hair cells in the cochlea. Furthermore, when the Myc and Notch 1 intracellular domains were transiently activated for 3 days, the adult supporting cells were able to proliferate and then, following Myc downregulation, transdifferentiate into hair cell (HC)-like cells through the induction signal of Atoh1. Therefore, the transient nature of Myc and Notch and their subsequent downregulation are vital for the trans-differentiation process (Shu et al., 2019), further highlighting the need for transient Myc expression in regeneration systems. More recently, in an attempt to generate a clinically applicable regenerative therapeutic of Myc and Notch overexpression, Quan et al. (2023) used a cocktail composed of small molecules and siRNAs injected into the middle ear space, following injury and demonstrated regeneration of HC-like cells in response to Atoh1. However, the regeneration efficiency was attenuated compared to that achieved by ectopic Myc expression from a transgenic allele in the mouse and suggests that optimisation of Myc expression is required.

In the optic nerve, retinal ganglion cells (RGCs) are vital for the propagation of visual information from the eye to the brain through projections of their axons that run along the optic nerve. Unlike zebrafish, that can restore vision via the dedifferentiation and proliferation of Müller glia cells that generate all cell types required to regenerate, mammals lose the ability to regenerate their RGCs shortly after birth. Upon injury of the optic nerve, apoptosis of RGCs leads to an irreversible loss of vision (Boia et al., 2020; Soucy et al., 2023). Interestingly, single-cell RNA sequencing has shown that Myc is expressed in certain RGC subtypes (Rheaume et al., 2018) and that the expression of Myc mRNA is decreased in the optic nerve by 70%, following injury (Belin et al., 2015). Recently, it was found that Myc regulates axonal regeneration in the sensory optic nerve through the downstream target, telomerase reverse transcriptase (TERT), and p53. Both TERT and p53 are upregulated following an injury and decrease in expression when the sensory axons mature and lose the ability to grow. The functional inhibition of TERT and p53 or Myc resulted in impairment in axonal regeneration (Ma et al., 2019). Knockout of Myc significantly reduced the number of regenerating axons, whilst overexpression of Myc in the RGCs of mice orchestrated increased survival that drove regeneration of their axons, following optic nerve injury. Furthermore, a synergistic effect of AAV-mediated Myc overexpression combined with the co-deletion of PTEN and SOCS3 promoted neuronal survival and axon regeneration. Interestingly, delayed overexpression of Myc to day 1 following injury, which is more clinically relevant, continued to demonstrate that Myc could still rescue and improve the survival of injured neurons and induce axonal regeneration. These regenerated axons were also found to grow outside the injury site, highlighting an exciting prospect for neuronal regeneration (Belin et al., 2015).

Oligodendrocyte progenitor cells (OPCs) are a subtype of proliferating glia in the CNS that differentiate into myelinating oligodendrocytes which support and insulate axons. Myc expression is elevated in proliferating OPCs, and Myc plays a key role in their maintenance in a proliferative and undifferentiated state. The level of Myc in OPCs declines during the differentiation into oligodendrocytes in the developing white matter (Magri et al., 2014). The ability of OPCs to proliferate and differentiate into oligodendrocytes becomes impaired with ageing, and there is an age-related decline in the efficiency of re-myelination which can contribute to the progression of neurological diseases such as multiple sclerosis (Sim et al., 2002; Kuhlmann et al., 2008; Boyd et al., 2013; Neumann et al., 2019; Neumann et al., 2021). There is a correlation between the age-related decline of OPC function and Myc expression whereby Myc levels have been shown to dramatically reduce over time during OPC ageing, suggesting that Myc plays a role in maintaining the identity of OPCs (Neumann et al., 2021). In agreement with this hypothesis, the inhibition of Myc in neonatal OPCs leads to a quiescent state and aged-like OPC characteristics, loss of OPC self-renewal capacity, and the ability to differentiate (Neumann et al., 2021). Conversely, restoring the proliferative capacity of OPCs aids the differentiation potential of OPCs and enhances re-myelination efficiency (Foerster et al., 2020). Therefore, Myc overexpression has been examined in aged OPCs, and ectopic Myc expression can revert OPCs to a more neonatal-like state characterised by increased proliferative potential whilst also increasing the ability to differentiate into myelinating oligodendrocytes. *In vivo*, the enhanced function of the aged OPCs by Myc showed an improvement in the myelin regeneration of the axons in aged animals where there is a poor re-myelination potential and efficiency due to their aged CNS. These results demonstrate that Myc can change the functional age of OPCs, highlighting a new strategy for treating neurological diseases such as multiple sclerosis, where the myelin sheath is damaged. However, in the case of OPCs, long-term Myc expression would likely be required to maintain OPCs in their proliferative, juvenile state, but given the oncogenic risk of gliomas it may be difficult to harness Myc directly (Neumann et al., 2021).

## 6 Concluding remarks

Myc was first discovered in the 1980s and has become one of the most extensively studied proteins, appearing in ∼50,000 publications listed on PubMed (1980–2024). Myc is a highly conserved protein across the animal kingdom that regulates many critical processes within cells. The expression of Myc is highly synchronized, and its expression is typically kept at low levels or restricted to highly proliferative tissues. Myc expression has been shown to be crucial for sustaining pluripotency (Fagnocchi and Zippo, 2017; Fan and Li, 2023) and is one of the four factors essential to efficiently reprogram adult somatic cells to induce pluripotent stem cells (iPSCs) (Takahashi and Yamanaka, 2006; Araki et al., 2011). The overexpression or deregulation of Myc is seen in the vast majority of all human cancers, and cancer cells share many molecular characteristics with iPSCs. This review has concentrated on evidence that Myc may be central to regenerative processes across species. Complex tissue regeneration requires the coordination of a series of fundamental biological processes, including, wound sensing, barrier formation, cell cycle re-entry, migration, trans-differentiation, and remodelling. These processes are characterized by the altered expression of transcription factors, temporary de-differentiation, and the loss of cell fate markers. In regenerative species and organs, these expression changes are temporary and generally revert to baseline following resolution of the injury. Endogenous reparative regeneration is an emerging field that aims to restore organ function by harnessing and enhancing endogenous repair mechanisms. The Myc gene family is uniquely situated to synergize upstream pathways into downstream cell cycle control (Figure 5) and to correspondingly suppress differentiation-specific genes to allow for trans-differentiation. The data presented here highlight the need for controlled, transient, localized delivery of Myc. Careful consideration is therefore needed when selecting a possible therapeutic strategy to enhance Myc expression.

There are valid concerns that the ectopic expression of deregulated Myc may cause off-target effects or even neoplastic transformation. Any factor that is involved in cell growth and proliferation is, in essence, a proto-oncogene, and other factors capable of reactivating proliferation, such as the activation of Yap or Wnt signalling, are also potently oncogenic when deregulated. However, these are exactly the proteins that are required to drive efficient proliferation in non-regenerative tissues, and the need for these pro-proliferative factors highlights the importance for the systems that drive expression to be transient. Where reversible or transient expression systems are employed, de-differentiation is shown to be reversible (Bywater et al., 2020; Chen et al., 2021). The reversibility of deregulated Myc has also been observed in cancers where the deactivation of ectopic Myc in pancreatic and lung cancers leads to the complete regression of tumorigenesis and restoration of the normal tissue architecture (Kortlever et al., 2017; Sodir et al., 2020). The key, therefore, is the deactivation of Myc following immediate repair to aid re-differentiation and the later stages of the reparative regenerative program.

Similarly, where the long-term expression of pro-proliferative factors via AAV delivery systems has been pre-clinically employed, side effects from the continued proliferation such as the de-differentiation of cardiomyocytes and arrhythmic episodes have reduced their success (Gabisonia et al., 2019). From a safety perspective, the use of constitutive expression viral systems will probably be unsuccessful, so transient technologies with rapid kinetics are essential. A system such as mRNA that has no issues surrounding genetic integration and which allows a more “physiologically normal” pulse of Myc with its naturally short protein half-life, as observed following acute damage in regenerative systems, should be employed.

It must be noted that Myc may be harmful in some tissues; for instance, as well as the tumorigenic effect of Myc in the liver, Myc can induce liver fibrosis (Gabisonia et al., 2019). Therefore, in addition to transient or switchable technologies, cell-specific systems to restrict Myc expression to the cell types of interest are vital. Cell-specific systems for mRNA expression are beginning to emerge (Magadum et al., 2020a; Qian et al., 2022; Kaseniit et al., 2023) and hold much promise for application in endogenous regeneration. Furthermore, lipid nanoparticle cell targeting that impedes the accumulation of nucleic acid in hepatocytes is a future possibility (Kularatne et al., 2022).

Here, we have concentrated on the role of Myc in direct cell cycle regulation. However, Myc possesses the ability to mediate a plethora of processes resulting in microenvironment, immune (Kortlever et al., 2017), and metabolic (Stine et al., 2015) changes which are dependent on tissue. For instance, where Myc is specifically expressed in oncogenic KRas-driven lung epithelial tumour cells, Myc expression leads to a reversible influx of VEGF-expressing macrophages, an exclusion of T cells, and rapid onset of angiogenesis. In oncogenic KRas-driven pancreatic tumours, specific epithelial Myc activation leads to an influx of macrophages, an exclusion of T cells, but also an influx of neutrophils and an increase in activated fibroblasts and deposition of desmoplasia (Kortlever et al., 2017; Sodir et al., 2020). Therefore, Myc expression may not only lead to intrinsic cell number restoration but may be able to tap into the resident regenerative programs of tissues which may be vital for regeneration. Metabolic reprogramming is a key hallmark of cancer that is mostly directly regulated by Myc (Stine et al., 2015) and facilitates the generation of biomass for rapid cell proliferation. Likewise, cellular metabolism plays a key role during regeneration. In the heart, loss of mammalian cardiac regenerative capacity correlates with an increased metabolic state (a metabolic switch from glycolysis to fatty acid oxidation). Mimicking these changes in ES-cell derived cardiomyocytes can drive cells to become more mature and proliferate less (Mills et al., 2017). Conversely, metabolic reprogramming can allow for cardiomyocyte proliferation and cardiac regeneration *in vivo* (Magadum et al., 2020b; Bae et al., 2021; Li et al., 2023). Therefore, during complex tissue regeneration, Myc may not only provide the stimulus for cell cycle but also the capability for the demands of growth and communication with the surrounding environment.
